# HIV-1 Vpr-Mediated G2 Arrest Involves the DDB1-CUL4A^VPRBP^ E3 Ubiquitin Ligase

**DOI:** 10.1371/journal.ppat.0030085

**Published:** 2007-07-13

**Authors:** Jean-Philippe Belzile, Ghislaine Duisit, Nicole Rougeau, Johanne Mercier, Andrés Finzi, Éric A Cohen

**Affiliations:** 1 Laboratory of Human Retrovirology, Institut de Recherches Cliniques de Montréal, Montreal, Quebec, Canada; 2 Department of Microbiology and Immunology, Université de Montréal, Montreal, Quebec, Canada; Oregon Health and Science University, United States of America

## Abstract

Human immunodeficiency virus type 1 (HIV-1) viral protein R (Vpr) has been shown to cause G2 cell cycle arrest in human cells by inducing ATR-mediated inactivation of p34cdc2, but factors directly engaged in this process remain unknown. We used tandem affinity purification to isolate native Vpr complexes. We found that damaged DNA binding protein 1 (DDB1), viral protein R binding protein (VPRBP), and cullin 4A (CUL4A)***—***components of a CUL4A E3 ubiquitin ligase complex, DDB1-CUL4A^VPRBP^ ***—***were able to associate with Vpr. Depletion of VPRBP by small interfering RNA impaired Vpr-mediated induction of G2 arrest. Importantly, VPRBP knockdown alone did not affect normal cell cycle progression or activation of ATR checkpoints, suggesting that the involvement of VPRBP in G2 arrest was specific to Vpr. Moreover, leucine/isoleucine-rich domain Vpr mutants impaired in their ability to interact with VPRBP and DDB1 also produced strongly attenuated G2 arrest. In contrast, G2 arrest–defective C-terminal Vpr mutants were found to maintain their ability to associate with these proteins, suggesting that the interaction of Vpr with the DDB1-VPRBP complex is necessary but not sufficient to block cell cycle progression. Overall, these results point toward a model in which Vpr could act as a connector between the DDB1-CUL4A^VPRBP^ E3 ubiquitin ligase complex and an unknown cellular factor whose proteolysis or modulation of activity through ubiquitination would activate ATR-mediated checkpoint signaling and induce G2 arrest.

## Author Summary

Human immunodeficiency virus type 1 (HIV-1), the causative agent of AIDS, encodes several proteins termed accessory, which play a critical role in viral pathogenesis. Collectively, HIV-1 accessory proteins Vif, Vpr, Vpu, and Nef have been shown to manipulate host cell biology to promote viral replication, immune evasion, and AIDS progression. In this context, Vpr has been found to block normal cell growth. This interruption of cell division by Vpr is thought to increase viral replication and induce programmed cell death. However, how Vpr is able to block cell division remains unknown. To understand this mechanism, we have studied the interaction between Vpr and novel cellular proteins. We identified a novel complex comprising Vpr and cellular proteins involved in a process called ubiquitination. Ubiquitination is a mechanism by which a small protein, ubiquitin, is conjugated to cellular proteins to modulate their activity or induce their degradation. We demonstrated that association of Vpr with this ubiquitinating complex might be responsible for the defect in cell growth. Further characterization of this protein complex and the elucidation of the mechanism by which it affects cell growth might lead to the development of new anti-HIV drugs and an improved understanding of the basic cellular processes controlling cell division.

## Introduction

Human immunodeficiency virus type 1 (HIV-1) viral protein R (Vpr) accessory protein is a small 96−amino acid protein that plays several roles during virus infection (reviewed in [1,2]). In particular, the protein mediates cell cycle arrest at the G2/M transition in various mammalian cells [3–6], a cytostatic ability that is well conserved among the primate lentiviruses [7]. Its biological significance is not fully understood but may be related to general activation of virus expression [8] and/or induction of apoptosis [9]. Vpr suppresses cell proliferation by preventing the activation of the p34cdc2/cyclin B complex [3,4]. Accumulating evidence indicates that Vpr-mediated cell cycle arrest depends on DNA damage response, but precise mechanisms of its induction remain obscure.

Phosphatidylinositol 3 kinase−like ATM (ataxia telangiectasia mutated) and ATR (ataxia and telangiectasia mutated and Rad3 related) are key components of the G2/M checkpoint. In addition, ATR activates S-phase checkpoint in replication stress response resulting from stalled replication [10]. Depending on the type of stress, ATR- or ATM-mediated checkpoints are fully activated by the coordinated activity of Rad9-Rad1-Hus1 (9-1-1), Rad17-RFC, breast cancer−associated protein (BRCA1), and p53 binding protein (53BP) (reviewed in [11–13]). ATR- or ATM-dependent phosphorylation of the histone variant H2AFX (H2AX) triggers the formation of γ-H2AFX/BRCA1 or 53BP foci. These foci are presumed to help in the recruitment and/or retention of DNA repair machinery and checkpoint effectors at the damaged DNA sites, thus promoting checkpoint signal amplification [12]. Downstream activation of CHEK1 or CHEK2 kinases by ATM and ATR results in the inactivation of Cdc25 phosphatase and increased expression of both WEE1 kinase and the 14-3-3 family of proteins. Inactivation of cdc2/cyclin by hyperphosphorylation and cytoplasmic retention prevents entry into mitosis before the completion of DNA repair [10,12].

Several groups have reported that Vpr expression induces Rad17- and Hus1-dependent activation of ATR, but not of ATM, and induces the formation of nuclear γ-H2AFX/BRCA1 foci [14–16]. However, the mechanism by which Vpr triggers ATR activation is not well understood. Some authors proposed that Vpr would interfere with normal DNA replication, leading to stalled replication forks [15,17], while others suggested that the protein may promote the formation of DNA double-strand breaks by recruiting unknown endonucleases to the chromatin [18]. Both models imply direct Vpr interactions with host chromatin [15,18]. On the other hand, blockade of the proliferation might rely on the mislocation of key cell cycle regulators, because of alterations in the nuclear envelope induced by membrane-anchored Vpr [19].

To identify Vpr-interacting cellular proteins responsible for the initial events leading to the induction of G2 arrest, we used the proteomic tandem affinity purification (TAP) procedure followed by mass spectrometry. Native complexes containing TAP-Vpr were purified from human cells by two consecutive affinity chromatographic steps under mild conditions. Here, we identify a novel protein complex comprising Vpr, the damaged DNA binding protein 1 (DDB1), the E3 ubiquitin ligase scaffold protein cullin 4A (CUL4A), and the newly identified DDB1-CUL4A–associated factor 1 (DCAF1), which is also designated as viral protein R binding protein (VPRBP) [20–22]. We provide functional evidence indicating that Vpr interaction with this E3 ubiquitin ligase complex is involved in induction of G2 cell cycle arrest.

## Results

### Vpr Interacts with DDB1 and VPRBP

To purify cellular protein complexes interacting with HIV Vpr, Vpr was fused to a TAP tag containing two immunoglobulin-binding domains of protein A from *Staphylococcus aureus,* a cleavage site for the tobacco etch virus (TEV) protease, and the calmodulin-binding peptide (CBP). Since Vpr C-terminal modifications have been reported to alter its cytostatic abilities [23], the bipartite tag was introduced N-terminally. Purification of TAP-Vpr–containing complexes was then conducted in human embryonic kidney (HEK) 293T cells, although the tagged protein induced cell cycle arrest less efficiently than wild-type Vpr (unpublished data). After electrophoresis and silver staining, two bands corresponding to high–molecular weight proteins were repeatedly observed (unpublished data). Matrix-assisted laser desorption/ionization time-of-flight mass spectrometric analysis revealed that the upper band corresponded to VPRBP, a 180-kDa protein that had been isolated as a Vpr-binding factor a decade ago, but whose function still remained obscure [21,22]. The other 120-kDa protein was identified as DDB1, initially considered part of a heterodimeric complex containing damaged DNA binding protein 2 (DDB2), involved in a cellular response to UV-induced DNA damages [24,25]. However, the protein is now emerging as a central scaffolding factor in the DDB1-CUL4A-RBX1 E3 ubiquitin ligase complex associated with the COP9 signalosome [26]. Importantly, recently the WD40 protein VPRBP has been demonstrated to interact with DDB1 and probably serves as an adapter to confer substrate specificity to the DDB1-CUL4A-RBX1 E3 ubiquitin ligase complex [20].

**Figure 1:**
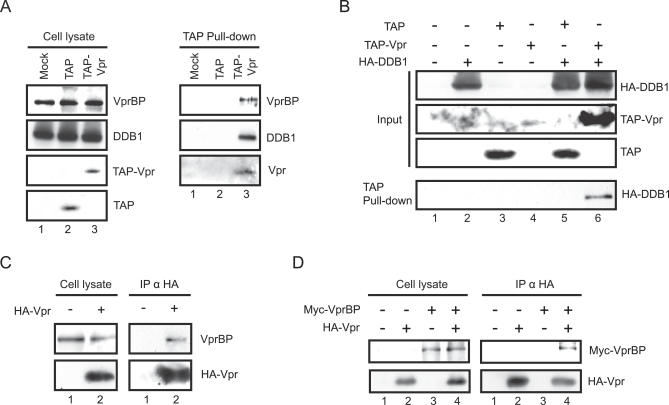
Immunoprecipitation of DDB1/Vpr and VPRBP/Vpr Complexes (A) HEK293T cells were mock transfected (lanes 1) or transfected with either TAP (lanes 2) or TAP-Vpr–expressing plasmids (lanes 3). Two days later, immunoprecipitations of TAP tag were performed on cell lysates using IgG-coupled beads and purified complexes were eluted by cleavage with TEV protease. The levels of endogenous VPRBP and DDB1 were monitored in crude lysates and pulled-down fractions by Western blot using specific antibodies. TAP, TAP-Vpr, and cleaved Vpr were detected using a polyclonal rabbit antibody directed against a Vpr N-terminal peptide. (B) HEK293T cells were mock transfected (lanes 1 and 2) or transfected with either TAP (lanes 3 and 5) or TAP-Vpr–expressing plasmids (lanes 4 and 6). Cells were transcomplemented with the empty vector (lanes 1, 3, and 4) or HA-DDB1–encoding plasmid (lanes 2, 5, and 6). (C) HEK293T cells were mock transfected (lanes 1) or transfected with HA-Vpr–expressing plasmid (lanes 2). Immunoprecipitations using anti-HA antibodies were performed on cell extracts using protein A–sepharose beads. The levels of HA-Vpr and endogenous VPRBP were monitored in cell extracts as well as immunoprecipitated fractions by Western blot using specific antibodies. (D) HEK293T cells were mock transfected (lanes 1 and 3) or transfected with a HA-Vpr–expressing plasmid (lanes 2 and 4). Cells were transcomplemented with the empty vector (lanes 1 and 2) or Myc-VPRBP–encoding plasmid (lanes 3 and 4). Anti-HA immunoprecipitations were performed as described above.

We sought to confirm the interaction of Vpr with DDB1 and VPRBP in HEK293T cells transfected with TAP or TAP-Vpr expression plasmids. TAP pull-down experiments were performed on cell lysates using IgG-coated sepharose beads. Co-precipitated endogenous DDB1 and VPRBP were detected by Western blot using specific antibodies. As shown in Figure 1A, endogenous DDB1 and VPRBP could be pulled down when co-expressed with TAP-Vpr (lane 3), but not when the protein was in the presence of the native TAP tag (lane 2), indicating that DDB1 and VPRBP binding was specific to TAP-Vpr. These interactions could be detected in conditions containing 1% NP40 (unpublished data) as well as 0.5% Triton X-100 (Figure 1A).

To confirm the specificity of the interaction between Vpr and DDB1, we performed pull-down assays in cells co-transfected with TAP-Vpr and hemagglutinin (HA)-tagged DDB1–encoding plasmids (Figure 1B). We were able to observe that HA-DDB1 could be co-precipitated specifically in the presence of TAP-Vpr (lane 6), but not in the presence of the empty plasmid (lane 2) or a TAP-expressing plasmid (lane 5). We constructed TAP-DDB1 as well as green fluorescent protein (GFP)–tagged DDB1 expression plasmids to verify whether the interaction could be observed in the reversed orientation. However, immunoprecipitation using endogenous, TAP-tagged, HA-tagged, or GFP-fused DDB1 as bait and wild-type or HA-tagged Vpr yielded inconsistent results (unpublished data). These discrepancies between HA-Vpr and TAP-Vpr abilities to bind to DDB1 are reminiscent of the versatile association between DDB1 and the DNA replication licensing factor CDT1. In that case, detection of DDB1-CDT1 complexes in absence of chromatin was dependent on the amount of antibody used for the immunoprecipitation [20]. Given that CDT1 interacts indirectly with DDB1 via the adapter protein CDT2 [27,28], it is tempting to hypothesize that Vpr would likewise interact indirectly with DDB1 through an adapter protein, perhaps VPRBP, and that the TAP/IgG bead complexes may somehow stabilize the interaction.

To confirm the specificity of interaction between Vpr and VPRBP in our system, we performed co-immunoprecipitation experiments in the presence of endogenous VPRBP and over-expressed HA-Vpr (Figure 1C). We could specifically detect co-immunoprecipitated VPRBP in the presence of HA-Vpr (lane 2), but not in the presence of the empty plasmid (lane 1). Finally, we observed an interaction between over-expressed HA-Vpr and Myc-tagged VPRBP (Figure 1D), confirming the specificity of the interaction between Vpr and VPRBP.

### DDB1 Appears to Bind to Vpr Indirectly

**Figure 2:**
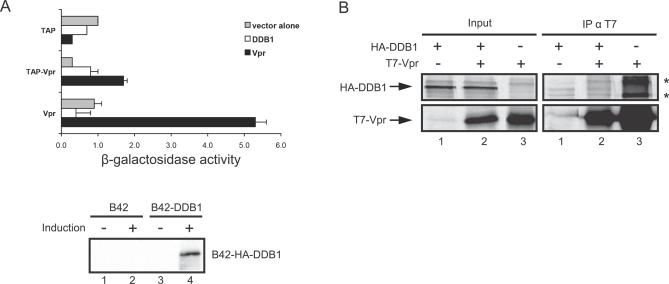
Absence of Direct Vpr Binding to DDB1 in Yeast (A) The EGY48 reporter strain containing LexA-TAP, LexA-Vpr, or LexA-TAP-Vpr (“bait”) was transformed with B42, B42-DDB1, or B42-Vpr–expressing plasmid (“prey”). The binding affinity between the different proteins was assessed by assaying β-galactosidase activity using the *o*-nitrophenyl-β-D-galactopyranoside method. Histograms represent averaged data from 2–4 different clones and are representative of two independent assays. Western blot analysis of induced and non-induced B42-HA-DDB1 expression in the B42 and B42-DDB1-transformed EGY48/LexA-Vpr reporter strain is shown below. (B) In vitro–translated T7-Vpr was immunoprecipitated with an anti-T7 antibody in the presence or absence of in vitro–translated HA-DDB1. Amounts of protein initially added to the assay (input) are shown in the left panel. * represents non-specific proteins immunoprecipitated by the anti-T7 antibody.

To further investigate the apparent association between TAP-Vpr and DDB1, Vpr, TAP, and TAP-Vpr sequences were subcloned in yeast two-hybrid expression constructs. Saccharomyces cerevisae cells were co-transformed with each combination of plasmids. Interaction affinities were determined by measurement of the β-galactosidase activity. As expected, Vpr appeared to form homodimers, leading to strong reporter gene activity (Figure 2A). Surprisingly, no binding could be detected between Vpr and DDB1, a result consistent with the lack of interaction observed with some co-immunoprecipitation experiments. We found that dimerization of TAP-Vpr was three times weaker than that of the wild-type protein. Hence, N-terminal addition of large peptide appears to disturb the tertiary structure of Vpr, explaining at least in part the reduction of TAP-Vpr cytostatic abilities. Importantly, we found that β-galactosidase activity remained undetectable in cells co-expressing TAP-Vpr and DDB1. This lack of stable interaction between Vpr and DDB1 did not result from lack of DDB1 expression in yeast, since we could observe DDB1 expression by Western blot in Vpr-expressing yeast cells (Figure 2A). Finally, an association between Vpr and DDB1 could not be detected in co-immunoprecipitation experiments using in vitro–translated T7-tagged Vpr and HA-DDB1 proteins (Figure 2B) in conditions that have demonstrated an association between Vpr and VPRBP [21]; this suggests that the binding observed in human cells might be indirect and may involve a bridging factor.

### Vpr Associates with a DDB1-CUL4A^VPRBP^ E3 Ubiquitin Ligase Complex

One possible explanation for the apparent association between Vpr and DDB1 is that Vpr would associate with the DDB1-CUL4A-RBX1 ubiquitin ligase complex through a direct interaction with VPRBP. Indeed, recently the WD40 protein VPRBP has been demonstrated to interact with DDB1 and probably serves as an adapter to confer substrate specificity to the DDB1-CUL4A-RBX1 E3 ubiquitin ligase complex [20].

**Figure 3:**
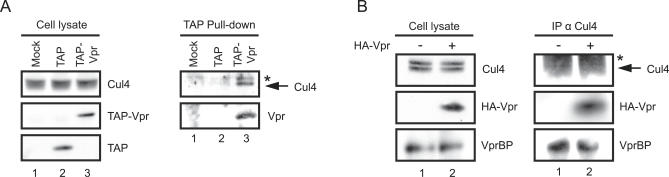
Vpr Associates with the Ubiquitin Ligase Scaffold Protein CUL4A (A) Five million HEK293T cells were transfected with 40 μg of empty (lanes 1), TAP-expressing (lanes 2), or TAP-Vpr–expressing plasmids (lanes 3). Forty-eight hours after transfection, TAP pull-downs were performed on cell lysates using IgG-coupled beads, and purified complexes were eluted by cleavage with TEV protease. The levels of endogenous CUL4A were monitored in crude lysates and pulled-down fractions by Western blot using a polyclonal goat anti-CUL4A antibody. TAP, TAP-Vpr, and cleaved Vpr were detected using a polyclonal rabbit antibody directed against a Vpr N-terminal peptide. (B) Ten million HEK293T cells were transfected with 80 μg of empty plasmid (lanes 1) or with HA-Vpr–expressing plasmid (lanes 2). Immunoprecipitation of endogenous CUL4A was performed using a goat polyclonal anti-CUL4A antibody and protein A–sepharose beads. The levels of endogenous CUL4A, VPRBP, and over-expressed HA-Vpr were monitored in crude lysates and immunoprecipitated fractions by Western blot using, respectively, a polyclonal goat anti-CUL4A antibody, a polyclonal rabbit anti-VPRBP antibody, and a monoclonal mouse anti-HA antibody. * represents a non-specific protein detected by the anti-CUL4A antibody. The anti-CUL4A antibody generally recognized a doublet of CUL4A when the gel resolution was sufficiently high. In the TAP pull-down fractions, only the upper band of CUL4A was detected.

To assess the possibility that the Vpr-VPRBP-DDB1 complex might be part of an ubiquitin E3 ligase complex, we investigated whether Vpr could associate with the E3 ligase scaffold protein CUL4A. HEK293T cells were transfected with plasmids expressing TAP and TAP-Vpr, and pull-down assays were performed on cell extracts containing endogenous CUL4A. These assays demonstrated that endogenous CUL4A could specifically associate with TAP-Vpr but not with native TAP (Figure 3A, lane 3), suggesting that Vpr associates with a CUL4A-scaffolded E3 ligase complex. Moreover, anti-CUL4A immunoprecipitation experiments on cells transfected with an empty plasmid or an HA-Vpr–expressing plasmid revealed that HA-Vpr co-immunoprecipitated with a CUL4A-containing complex (Figure 3B, lane 2), confirming that Vpr can associate with an E3 ligase complex with potential ubiquitinating activities. Importantly, VPRBP could be co-immunoprecipitated along with CUL4A (Figure 3B). Therefore, given the mutual association of Vpr (Figure 1A, 1C, and 1D therein and [21,22]) and CUL4A (Figure 3B therein and [20]) with VPRBP, these results suggest that Vpr interacts with the DDB1-CUL4A^VPRBP^ E3 ubiquitin ligase in a single complex and that this association might occur via the intermediary of VPRBP.

### The Interaction between DDB1-CUL4A^VPRBP^ and Vpr Is Required for the Induction of G2 Arrest

**Figure 4:**
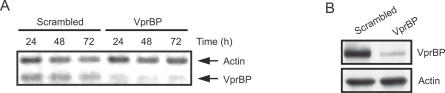
Depletion of VPRBP Using siRNA HEK293 cells were transfected with 300 pmol of VPRBP-targeting siRNA or control scrambled siRNA using Oligofectamine. (A) At 24, 48, and 72 h post-transfection, RNA was extracted and analyzed by RT-PCR to determine the extent of VPRBP downregulation at the mRNA level. PCR products were analyzed in the exponential phase of amplification. Actin levels were used as a control for RNA quality and reverse transcription efficiency. (B) Forty-eight hours after siRNA transfection, cells lysates were harvested and analyzed by Western blot using a polyclonal rabbit anti-VPRBP antibody to demonstrate the downregulation of VPRBP at the protein level. Actin levels were used as a control for protein loading.

**Figure 5:**
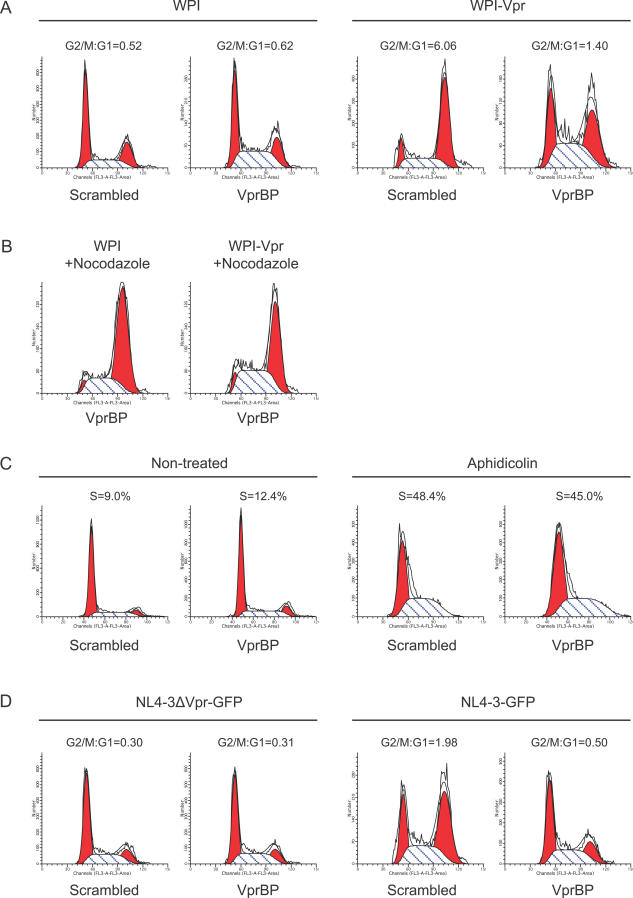
Effect of VPRBP Depletion on Vpr-Induced G2 Arrest (A) HEK293 cells were transfected with 300 pmol of VPRBP-targeting siRNA or control scrambled siRNA using Oligofectamine, followed by the same transfection 24 h later. Twenty-four hours after the second siRNA transfection, cells were transduced at a multiplicity of infection of 1 with lentiviral vectors expressing Vpr (WPI-Vpr) or the empty vector (WPI). Cell cycle profiles were analyzed 24 h after transduction by flow cytometry using propidium iodide staining. (B and C) To determine if cell growth (B) or checkpoint activation (C) was affected by VPRBP knockdown, HEK293 cells were transfected once with siRNA, as described above, and treated respectively with 1 μg/ml nocodazole and 0.5 μM aphidicolin 24 h later. Cell cycle profiles were analyzed 24 h after drug treatment. (D) To determine if VPRBP knockdown could also abrogate the induction of G2 arrest in the context of viral infection, siRNA-transfected cells were infected with NL4.3-GFP and NL4–3ΔVpr-GFP at a concentration of 100 cpm/cell and cell cycle profiles were analyzed 48 h later. Percentages of G1 and G2/M cell populations were determined using the ModFit software. These results are representative of the data obtained in at least two independent experiments.

We next sought to study the potential role of DDB1-CUL4A^VPRBP^ E3 ubiquitin ligase in the induction of G2 cell cycle arrest by Vpr. DDB1 depletion by small interfering RNA (siRNA) has been reported to induce the accumulation of cells in G2/M due to DNA re-replication [28]; consequently, this strategy could not be used to demonstrate the involvement of DDB1-CUL4A^VPRBP^ in the induction of Vpr-mediated G2 arrest. As an alternative strategy, we analyzed the effect of siRNA-mediated VPRBP knockdown on Vpr cytostatic properties. Cells transfected with VPRBP siRNA displayed a major reduction of VPRBP at the mRNA level (Figure 4A) as well as at the protein level (Figure 4B) compared with scrambled siRNA-transfected cells. We thus transfected HEK293 cells with siRNA against VPRBP and, 24 h later, transduced these cells with a lentiviral vector co-expressing GFP and native Vpr. We observed that cells transfected with VPRBP siRNA displayed strongly attenuated Vpr-mediated G2 arrest as compared with cells that received scrambled control siRNA (Figure 5A). This difference in the induction of G2 arrest was not due to a defect in infectivity potentially resulting from VPRBP knockdown, because the levels of transduction were equivalent in all the conditions tested (unpublished data). To verify that this defect in the induction of G2 arrest in VPRBP-depleted cells was the result of the abrogation of the Vpr–VPRBP interaction rather than a defect in cell growth, we treated these cells with nocodazole with the rationale that properly cycling cells should accumulate in mitosis because of the effect of the drug on microtubule polymerization (Figure 5B). In response to nocodazole, cells transfected with VPRBP siRNA alone or with the concomitant expression of Vpr accumulated at the G2/M phase, indicating that knockdown of VPRBP specifically impaired Vpr-mediated G2 arrest functions. Moreover, the knockdown of VPRBP did not produce any observable defects in the activation of the ATR-mediated checkpoints, since treatment with low concentrations of aphidicolin, a DNA replication inhibitor known to produce DNA double-strand breaks at fragile chromosomal sites and to activate the ATR-mediated intra-S checkpoint [29–31], blocked cell cycle progression at the S-phase in scrambled as well as VPRBP siRNA-transfected cells (Figure 5C). Finally, we analyzed the role of VPRBP in the induction of G2 arrest by Vpr in the context of viral infection. SiRNA-transfected cells were infected with vesicular stomatatis virus glycoprotein G (VSV-G)–pseudotyped fully infectious isogenic viruses defective (NL4–3ΔVpr-GFP, *vpr−*) or proficient (NL4–3-GFP, *vpr+*) for Vpr expression. Cells were analyzed for their cell cycle profile 48 h after infection. As expected, cells transfected with scrambled control siRNA accumulated in G2 after infection with the *vpr+* but not with the *vpr−* virus (Figure 5D). Knockdown of VPRBP almost abrogated the accumulation of cells in G2 in response to *vpr+* virus infection, but did not significantly affect the cell cycle profile of *vpr−* virus–infected cells. Again, these differences in G2 arrest were not the result of differential infectivity (unpublished data). Overall, these results indicate that VPRBP is necessary for Vpr-induced G2 arrest, suggesting that the DDB1-CUL4A^VPRBP^ E3 ligase complex might be involved in this Vpr biological activity.

**Figure 6:**
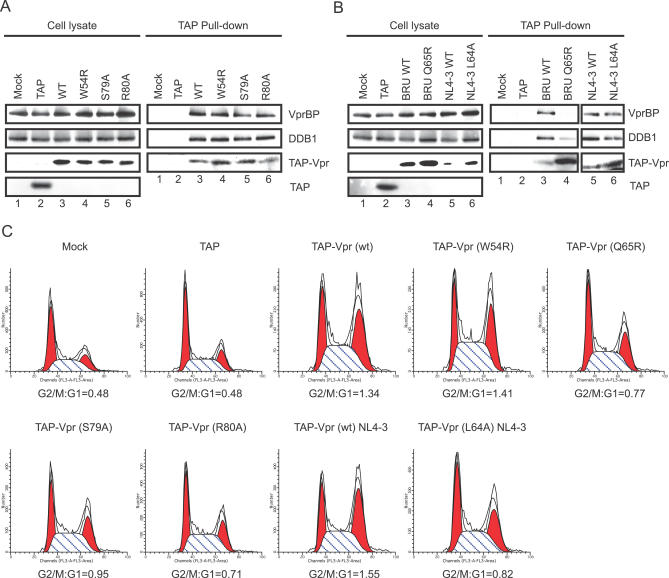
DDB1 and VPRBP Binding Affinities of TAP-Tagged Vpr Mutants (A) HEK293T cells were transfected with TAP-Vpr plasmids encoding for wild-type Vpr (lanes 3) or Vpr mutants W54R (lanes 4), S79A (lanes 5), and R80A (lanes 6). As control, cells were mock transfected (lanes 1) or transfected with a TAP-expressing plasmid (lanes 2). Following TAP pull-down using IgG-coupled beads, the levels of endogenous VPRBP and DDB1 were monitored in crude lysates and pulled-down fractions by Western blot using specific antibodies. TAP, TAP-Vpr, and cleaved Vpr were detected using a polyclonal rabbit antibody directed against a Vpr N-terminal peptide. (B) HEK293T cells were transfected with TAP-Vpr plasmids encoding for wild-type Bru Vpr (lanes 3) and wild-type NL4–3 Vpr (lanes 5), or Vpr mutants Bru Q65R (lanes 4) and NL4–3 L64A (lanes 6). As control, cells were mock transfected (lanes 1) or transfected with a TAP-expressing plasmid (lanes 2). TAP pull-downs and immunodetection of VPRBP, DDB1, TAP, and Vpr were performed as described for (A). (C) HEK293T cells were co-transfected with 1 μg of GFP-expressing plasmid and 15 μg of TAP-Vpr plasmids expressing wild-type or mutant proteins. Cell cycle analysis was performed using propidium iodide staining on the GFP+ cell population as described in Materials and Methods. Percentages of G1 and G2/M cell populations were determined using the ModFit software.

To examine whether the association of Vpr to the E3 ligase complex is required, we tested the ability of several TAP-tagged Vpr mutants to associate with VPRBP and DDB1 (Figure 6A and 6B) and assessed their effect on Vpr-induced G2 arrest (Figure 6C). HEK293T cells were transfected with plasmids expressing mutants of TAP-Vpr, and TAP pull-down experiments were performed on these transfectant cellular extracts. As previously observed, TAP-Vpr was able to pull down endogenous VPRBP and DDB1 (Figure 6A, lane 3). Interestingly, the classical Vpr mutants S79A and R80A that are attenuated for the induction of G2 arrest (Figure 6C; [32,33]) could still associate with VPRBP and DDB1 at levels comparable with that of the wild-type protein (Figure 6A, compare lane 3 with lanes 5 and 6), suggesting that the association between Vpr and the DDB1-VPRBP complex, per se, is not sufficient to block cell cycle progression. Moreover, W54R, a mutant of Vpr that was previously shown to be defective for the interaction and degradation of uracil-DNA glycosylase (UNG2/CCNU) [34], was still capable of associating with the DDB1-CUL4A^VPRBP^complex (Figure 6A, lane 4) and mediating G2 arrest (Figure 6C), suggesting that UNG2 and VPRBP bind to two distinct domains on Vpr. Zhao et al. previously mapped the domain of interaction of VPRBP to the leucine/isoleucine–rich domain of Vpr [22]. We analyzed whether the L64A and Q65R mutations in this domain of Vpr could abrogate binding to VPRBP. Using our TAP-Vpr pull-down assay, we observed a very strong reduction of binding to VPRBP and DDB1 with the Q65R mutant (under longer exposure VPRBP could be detected) (Figure 6B, compare lanes 3 and 4). A residual association with DDB1 was observed under these conditions, most likely reflecting difference in antibody affinities. Additionally, the reduction of TAP-Vpr binding to VPRBP and DDB1 could be observed with the L64A mutant, though it was less pronounced (Figure 6B, compare lanes 5 and 6). Interestingly, with both mutants, the reduced affinity for VPRBP was accompanied by a concomitant decrease in the association with DDB1, again suggesting that VPRBP and DDB1 are likely to form a single complex with Vpr (Figure 6B, compare lane 3 with lane 4 and lane 5 with lane 6). Importantly, we found that Vpr (L64A) and Vpr (Q65R) were strongly attenuated for the induction of G2 arrest (Figure 6C). Indeed, the residual level of G2 arrest observed with these two mutants was comparable with the attenuated G2 arrest produced by the R80A mutation. Together, these results suggest that the interaction of Vpr with the DDB1-CUL4A^VPRBP^ E3 ubiquitin ligase complex is necessary but not sufficient to induce G2 arrest.

## Discussion

The induction of G2 arrest by the HIV-1 accessory protein Vpr was described more than ten years ago [3–6]; however, the mechanism by which Vpr can accomplish this function has remained elusive. Several recent reports have demonstrated that expression of Vpr leads to activation of ATR, as well as to the formation of DNA repair foci containing BRCA1 and γ-H2AFX [14–16]. Nonetheless, the initial events leading to ATR checkpoint signaling are not known. Herein, we have used the TAP method to isolate cellular protein complexes interacting with HIV-1 Vpr to identify cellular factors that would be involved in Vpr-mediated ATR activation and subsequent G2 arrest. Using this strategy, we have identified DDB1 and VPRBP as cellular factors forming a complex with Vpr.

DDB1 is part of the DDB1-CUL4A-RBX1 E3 ubiquitin ligase complex that targets proteins for degradation via the COP9 signalosome [26]. In this complex, DDB1 serves as a scaffold protein presenting substrate to the E3 ubiquitin ligase. The protein is structurally complex and contains three seven-bladed β propellers (βPA, βPB, and βPC) [35]. The βPB propeller is involved in the interaction with CUL4A, whereas the βPA–βPC double-propeller fold is responsible for substrate presentation via interaction with WD40-repeat proteins [35]. Over 15 different WD40-containing substrate receptors, including DDB2, CSA, DET1-COP1, and CDT2, have been shown to interact with DDB1 and are thought to confer substrate specificity [20,35–37]. However, to date, only a few cellular proteins have been found to be regulated by the DDB1-CUL4A-RBX1 complex. In human cells, DDB1-CUL4A^DDB2^ promotes the ubiquitylation of histone 2A [38], histone 3, histone 4 [39], and the xeroderma pigmentosum group C protein (XPC) to regulate their activity [40]. In contrast, DDB1-CUL4A^CSA^ and DDB1-CUL4A^DET1-COP1^ promote proteolysis of Cockayne syndrome type B gene product (CSB) [26] and c-JUN [41]. Recently, DDB1, via the WD40 adapter CDT2, has been shown to prevent DNA re-replication during normal S-phase progression or in response to S-phase accumulation of DNA lesions by regulating the degradation of the replication licensing factor CDT1 [27,28].

Interestingly, DDB1 also forms complexes with two other viral proteins, namely hepatitis B virus X protein [42–44] and simian paramyxovirus SV5 V protein [45,46]. Whereas the mechanisms underlying the DDB1-dependent cytotoxicity induced by hepatitis B virus X protein remain poorly understood, it has been established that SV5 V protein facilitates the ubiquitination and subsequent signalosome-mediated degradation of STAT1 [45]. Several data argue in favor of a functional involvement of DDB1-CUL4A-RBX1 in Vpr functions. CUL4A has been implicated in Vpr-induced degradation of UNG2 and SMUG1 proteins [47], and Vpr was shown to interact with VIP/mov34/CNS6, one of the subunits of CUL4A-associated signalosome [48].

In our system, an interaction between Vpr and DDB1 was observed when Vpr was fused to a TAP tag (TAP experiments, Figure 1A and 1B). However, co-precipitations in the reverse direction with DDB1 fused to other tags (TAP, HA, or GFP) only yielded inconsistent interaction results (unpublished data). The lack of apparent interaction between DDB1 and Vpr in that context may be due to our experimental conditions or to the type of association that engages Vpr and DDB1. In that regard, the lack of interaction between Vpr and DDB1 in the yeast two-hybrid system (Figure 2A) as well as in in vitro co-precipitation experiments (Figure 2B) argued for an indirect interaction between these two proteins, though definite demonstration of this will require a more thorough analysis of this association in the future. These results were in contrast to SV5 V protein, which can directly interact with the DDB1 βPA–βPC domain [35,49]. Through the use of TAP procedures (unpublished data), immunoprecipitation experiments (Figure 1), and several Vpr mutants (Figure 6), we identified VPRBP as a cellular factor also in a complex with Vpr and DDB1 that possibly permits the recruitment of a DDB1-CUL4A E3 ubiquitin ligase complex. The cellular function of VPRBP has recently been uncovered through different proteomic approaches. It is a WD40 protein linked to the DDB1-CUL4A-RBX1 complex and probably serves as an adapter presenting protein substrate for degradation [20]. Interestingly, no VPRBP ortholog has been identified in yeast [21], perhaps explaining why we did not observe an interaction between Vpr and human DDB1 in S. cerevisae (Figure 2A) and why the DDB1 ortholog was not found among the putative Vpr-interacting proteins isolated by TAP assay in Schizosaccharomyces pombe (unpublished data).

To further characterize the possibility that Vpr might interact with a DDB1-CUL4A^VPRBP^ E3 ligase complex, we investigated whether the scaffold protein CUL4A could associate with Vpr. Interestingly, we could demonstrate the formation of a complex containing Vpr and CUL4A using two different approaches: by TAP-Vpr pull-down (Figure 3A) and by anti-CUL4A immunoprecipitation (Figure 3B). Therefore, it appears that Vpr can indeed recruit an E3 ligase complex with potential ubiquitinating activity. In these experiments, VPRBP was found in respective association with Vpr (TAP experiments, Figure 1A, 1C, and 1D), as well as with CUL4A (Figure 3B). Considering these interaction results and previous reports demonstrating the association between Vpr and VPRBP [21,22] as well as the association between CUL4A and VPRBP [20], it is most likely that Vpr interacts with the DDB1-CUL4A E3 ligase complex via the intermediary of VPRBP, although direct proof will require further analysis of the protein complex architecture.

Importantly, we observed that not only VPRBP but also the formation of a Vpr-DDB1-CUL4A^VPRBP^ complex was required for Vpr-mediated induction of G2 arrest. Indeed, siRNA targeting VPRBP strongly impaired the induction of G2 arrest in the context of a lentiviral vector expressing Vpr and of a fully infectious provirus (Figure 5A and 5D). Knockdown of VPRBP did not produce cell cycle aberrations (Figure 5B and 5C) or reduce viral/vector infection efficiency, suggesting that the observed defect in G2 arrest was specific to the association of Vpr with VPRBP.

It has been extensively shown that Vpr C-terminal residues 78−96 are important for G2 arrest [23,50–52], but we found that TAP-tagged Vpr mutants S79A and R80A attenuated for cell cycle arrest were nevertheless competent for association with DDB1 (Figure 6). This was not surprising given that Zhao et al. showed that VPRBP binding required the leucine/isoleucine-rich domain of Vpr [22]. In fact, L64A and Q65R, two mutations in the leucine/isoleucine-rich domain, reduced to different extents the binding of Vpr to VPRBP (Figure 6B). We also observed a concomitant decrease of affinity between Vpr and DDB1 as a result of these mutations, suggesting that a single complex comprised of VPRBP and DDB1 is binding to the leucine/isoleucine-rich domain of Vpr. Importantly, these two mutations strongly attenuated Vpr-induced G2 arrest (Figure 6C). Overall, these results suggest that recruitment of DDB1-CUL4A^VPRBP^ by Vpr is necessary but not sufficient to induce G2 cell cycle arrest.

One possible model to explain how Vpr induces G2 arrest via the DDB1-CUL4A^VPRBP^ complex is that Vpr could cause a generalized defect in the activity of the whole DDB1 complex. In this context, other investigators have shown that abrogation of DDB1 function using siRNA resulted in the failure to degrade CDT1, thus leading to the accumulation of re-replicated DNA fragment and the induction of an ATR-dependent G2 arrest [28]. Therefore, Vpr, through a potential sequestration of DDB1, might be capable of inducing the defective regulation of CDT1 or other DDB1-CUL4A E3 ligase complex substrates leading to ATR-mediated G2 arrest. However, we did not observe any obvious effect of Vpr on the S-phase degradation of CDT1 (unpublished data), which suggests that the DDB1-CUL4A-RBX1 complex was fully functional. Another possible model is that Vpr could block the proper ubiquitination of natural substrates of DDB1-CUL4A^VPRBP^, thereby affecting their biological activities or preventing their degradation. However, this possibility is unlikely because siRNA-mediated knockdown of VPRBP did not produce G2 arrest (Figure 5). The most probable model is that Vpr triggers the degradation of a yet-unknown modulator of cell cycle progression by targeting it to the substrate receptor VPRBP, itself linked to the DDB1-CUL4A-RBX1 E3 ligase complex. This situation would be highly similar to the mechanism by which Vpu induces the degradation of CD4 by recruiting directly CD4 to the SCF ^β-TRCP^ E3 ubiquitin ligase complex [53]. However, we cannot exclude the possibility that the Vpr–VPRBP interaction might modulate the activity of substrates through ubiquitination, as was described for XPC and histones [38–40]. Therefore, the formation of a complex between Vpr and DDB1-CUL4A^VPRBP^ through the Vpr leucine/isoleucine-rich domain could permit the hypothetical interaction of Vpr through its C-terminal domain with other cellular factors whose ubiquitination would induce the initial events leading to cell cycle arrest. This model is consistent with recent reports [54,55] demonstrating the involvement of VPRBP and DDB1 in Vpr-induced G2 cell cycle arrest.

In conclusion, we presented biochemical and functional evidence suggesting that Vpr is likely the third protein encoded by HIV-1, besides Vif and Vpu, to interact with the ubiquitination machinery. Vpr was found to associate with DDB1 and CUL4A, which are components of the DDB1-CUL4A-RBX1 E3 ligase complex, possibly through the intermediary of the WD40 substrate receptor VPRBP. Importantly, the interaction between Vpr and the VPRBP-DDB1 complex was shown to be required for the induction of G2 arrest. Vpr could act as a connector, bridging the DDB1-CUL4A^VPRBP^ E3 ubiquitin ligase to an unknown cellular factor whose proteolysis or modulation of activity through ubiquitination would activate ATR-mediated checkpoint signaling and cause G2 arrest.

## Materials and Methods

### Cells and antibodies.

HEK293 and HEK293T cells were maintained as described elsewhere [56]. The anti-HA tag and anti-Myc tag monoclonal antibodies were clones 12CA5 and 9E10, respectively. Rabbit polyclonal antibody against VPRBP was distributed by Accurate Chemical and Scientific Corporation (http://www.accuratechemical.com/). The mouse monoclonal antibody against DDB1 was obtained from BD Biosciences (http://www.bdbiosciences.com/). The goat polyclonal antibody against CUL4A was obtained from Santa Cruz Biotechnology (http://www.scbt.com/). Vpr was detected using a rabbit polyclonal antibody directed against a Vpr N-terminal peptide [57].

### Plasmid constructions.

For the construction of mammalian expression plasmid psvCMV-TAP, N-terminal TAP tag was first PCR amplified from the pBS1479 plasmid [58] using primers 5′-TCTAGACATATGGCAGGCCTTGCGCAAC-3′ and 5′-GGATCCTCACTACTCGAA-TCGTCATCATCAAGTGCC-3′, the last one containing two stop codons between the XhoI and BamHI restriction sites. The XbaI/BamHI-digested PCR fragment was then inserted into psvCMV plasmid [56] linearized with XbaI/BglII. For the construction of the psvCMV-TAP-Vpr expression plasmid, wild-type and mutant Vpr genes were PCR-amplified from the respective psvCMV-HA.Vpr plasmids [56] using oligonucleotides 5′-CTCGAGATGGAACAAGCCCCAG-3′ and 5′-GGATCCCTAGGATCTACTGGCT-3′. XhoI/BamHI Vpr fragments were finally fused to the XbaI/XhoI TAP sequence within psvCMV by 3-fragment ligation. Mammalian expression plasmids pSRAS-3HADDB1 and pSRAS-GFP.DDB1 [44] were kindly provided by M. Strubin (University of Geneva, Geneva, Switzerland). The TAP-DDB1 expression plasmid was constructed by inserting the XbaI/XhoI TAP fragment in Xba/SalI linearized pSRAS-3HA.DDB1 plasmid. The Myc-VPRBP expression plasmid was constructed by PCR amplification of the VPRBP cDNA from Image clone ID 5547856 (American Type Culture Collection, http://www.atcc.org/). The resulting PCR product was then subcloned in pCMV-Myc (Clontech, http://www.clontech.com/) at the SalI and NotI sites. For the construction of the yeast expression plasmids, TAP and TAP-Vpr sequences were subcloned after PCR amplification in the BamHI sites within pEG202. The DDB1 sequence was extracted from pSRAS-3HADDB1 by SalI/NotI digestion and introduced in pJG4–5. Vpr-expressing pJG4–5 and pEG202 plasmids have been described previously [59].

The second-generation self-inactivating lentiviral vectors pWPI and pWPXL, as well as the packaging plasmid psPAX2 were obtained from D. Trono (School of Life Sciences, Swiss Federal Institute of Technology, Lausanne, Switzerland). A lentiviral vector tranducing Vpr and GFP (pWPI-Vpr) was generated from pWPI. Vpr was PCR-amplified from psvCMV-Vpr [56], using primers 5′AAGGATCCATGGAACAAGCCCCAGAAGACC-3′ and 5′-TACGACTAGTCTAGGATCTACTGGCTCCATTT-3′, which contain a BamHI site at the 5′ end and a SpeI site at the 3′ end, respectively. The BamH1- and SpeI-cleaved PCR product was then ligated into pWPXL linearized at the same sites. A fragment containing the Vpr-coding sequence and the EF1-alpha promoter was excised with SpeI (Klenow-treated) and NotI and then ligated into pWPI at the PmeI and NotI sites to yield pWPI-Vpr.

### Tandem affinity purification.

Purification was done according to previously published procedures [58]. Briefly, HEK293T cells were seeded onto five to ten 150 mm-diameter plates. The next day, cells were transfected with 10 μg of psvCMV TAP-Vpr plasmid. Cells were collected 48 h later, washed, and lysed in IPP150 buffer (10 mM Tris-Cl [pH 8.0], 150 mM NaCl, 0.1% NP40) supplemented with EDTA-free complete protease inhibitors (Roche Diagnostics Canada, http://www.rochediagnostics.ca/). Cell debris was removed by low-speed centrifugation, and cleared supernatants were loaded onto IgG sepharose columns (Amersham BioSciences Canada, http://www.gelifesciences.com/). After extensive washes with IPP150 buffer and a final wash with TEV cleavage buffer (10 mM Tris-Cl [pH 8.0], 150 mM NaCl, 0.1% NP40, 0.5 mM EDTA, 1 mM DTT), TAP-containing complexes were eluted by overnight digestion using 100 units of TEV protease (Invitrogen Canada, http://www.invitrogen.com/) diluted in 1 ml of TEV buffer. Columns were eluted and washed with 3 ml of IPP150-calmodulin buffer (10 mM β-mercaptoethanol, 10 mM Tris-Cl [pH 8.0], 150 mM NaCl, 1 mM Mg-acetate, 1 mM imidazole, 2 mM CaCl_2_, 0.1% NP40) supplemented with 3 ml of 1 M CaCl_2_ and EDTA-free complete protease inhibitors. Fractions were passed through calmodulin columns (Stratagene, http://www.stratagene.com/), washed in IPP150 buffer, and finally eluted with a minimal volume of IPP150 elution buffer (10 mM β-mercaptoethanol, 10 mM Tris-Cl [pH 8.0], 150 mM NaCl, 1 mM Mg-acetate, 1 mM imidazole, 2 mM EGTA, 0.1% NP40). Recovered proteins were resolved by denaturing 12.5% SDS-PAGE. Bands detected after silver staining were cut and sent to Taplin Biological Mass Spectrometry Facility (Harvard Medical School, Boston, Massachusetts, United States) for MALDI-TOF.

### Transfection and immunoprecipitation.

Cells were transfected using the calcium phosphate precipitation method. Cells were harvested 2 d later, washed, and lysed in Triton lysis buffer (50 mM Tris-HCl [pH 7.5], 150 mM NaCl, 0.5% Triton X-100, and EDTA-free complete protease inhibitors). TAP pull-down assays were performed using 10 μl of IgG-coupled beads. After extensive washes in Triton lysis buffer, beads were resuspended in 100 μl of TEV cleavage buffer with 2 units of TEV and incubated for 16 h at 4 °C. Cleaved proteins were diluted in Laemmli buffer, heat-denatured, and loaded onto 12.5% acrylamide gels for SDS-PAGE. After protein transfer onto Hybond-ECL membrane (Amersham BioSciences Canada), proteins were detected by Western blot using specific antibodies. Immunoprecipitation experiments were performed on cell extracts lysed in the Triton lysis buffer as described previously [60].

### In vitro translation.

T7-Vpr and HA-DDB1 were in vitro translated using the Active Pro In Vitro Translation kit (Ambion, http://www.ambion.com/) according to manufacturer instructions.

### Cell cycle analyses.

Cell cycle analysis of HEK293 cells transduced by lentiviral vectors was performed as previously described in [56]. The mathematical model MODFIT was used to calculate the proportions of cells in the G2/M phases and G1 phase of the cell cycle. For analysis of cell cycle profile in the context of TAP-Vpr, HEK293T cells were co-transfected with 1 μg of a GFP-expressing plasmid and 15 μg of TAP-Vpr–expressing plasmids. Forty-eight hours after transfection, cells were fixed with 1% paraformaldehyde for 15 min followed by fixation/permabilization with 70% ethanol for 10 min. The rest of the procedure was as described previously [56] except that flow cytometry analysis was performed on the GFP-positive cell population.

### Yeast two-hybrid assay.

The yeast “bait” strains were made by transforming the EGY48 yeast strain with a URA3 lacZ (β-galactosidase) reporter plasmid and the different bait plasmids (expressing the HIS3 gene) by the lithium acetate method. The yeast “bait” strains harboring the bait and reporter plasmids were transformed with different prey plasmids and selected for the tryptophan autotrophy phenotype (in addition to the His and Ura nutritional markers for the bait and LacZ reporter plasmids, respectively). Determination of the respective interactions was performed as previously described [59].

### Lentiviral vector production, titration, and transduction.

VSV-G–pseudotyped viral particles were produced by transient transfection of 40 μg of vector (pWPI or pWPI-Vpr), 30 μg of the packaging construct psPAX2, and 12 μg of the VSV-G–expressing plasmid psvCMV-IN-VSV-G in five million HEK293T cells using the calcium phosphate precipitation method. The vector-containing supernatant was harvested 48 and 72 h post-transfection, 0.45 μm–filtered, and concentrated by ultracentrifugation on a sucrose cushion. Concentrated vectors were resuspended in culture medium and stored at −80 °C. Vectors were titered as described previously with some modifications [61]. Briefly, 5 × 10^4^ HEK293T cells were transduced with serial dilutions of the vector preparations in absence of polybrene. Twenty-four hours later, cells were fixed with 2% PFA for 30 min and the percentage of GFP-expressing cells was determined by flow cytometry. The vector titer was calculated as the number of transduction units in the linear range of transduction (5%–10% of GFP-positive cells). For transduction experiments, 1 × 10^5^ HEK293 cells seeded in the wells of a 6-well plate were incubated for 24 h with WPI or WPI-Vpr vectors (at a multiplicity of infection of 1) in presence of 8 μg/ml polybrene, typically achieving a transduction efficiency of 90%–95%. Cells were harvested after 24 h for flow cytometry analysis of cell cycle profiling and GFP expression.

### Viral clones and infection.

The infectious molecular clones pNL4.3-GFP and pNL4–3ΔVpr-GFP were obtained from Juan Lama (Department of Medicine, University of California San Diego, La Jolla, California, United States) and described in [62]. Virus was produced by transfecting 5 × 10^6^ HEK293T cells with 40 μg of proviral DNA and 10 μg of the VSV-G–expressing plasmid psvCMV-IN-VSV-G as described previously [56]. Virus-containing supernatant was titered by a standard RT assay as previously described [56]. Virions, at a concentration of 100 cpm/cell, were used to infect HEK293 cells in 1 ml of culture medium, in absence of polybrene. Forty-eight hours after infection, cells were harvested for flow cytometry analysis of cell cycle profiling and GFP expression.

### siRNA-mediated protein knockdown.

siRNA targeting VPRBP (siGenome SMARTpool M-021119–00) and scrambled control siRNA (non-targeting siRNA #2) were obtained from Dharmacon (http://www.dharmacon.com/). siRNA was transfected using Oligofectamine (Invitrogen Canada) according to the manufacturer instructions. Briefly, 300 pmol of siRNA were pre-incubated with 15 μl of Oligofectamine and overlayed on cells at 50% confluence (the final concentration of siRNA was 125 nM). Cells were transduced with lentiviral vectors 24 h after transfection. In some experiments, cells were subjected to two sequential transfections of 300 pmol of siRNA, 24 h apart.

### Reverse transcriptase polymerase chain reaction (RT-PCR).

RNA was extracted from siRNA-transfected cells at the given time using the Qiagen RNeasy kit (Qiagen, http://www.qiagen.com/) according to the manufacturer instructions. After elution, RNA was stored at −80 °C. All reagents for RT-PCR were purchased from Invitrogen Canada. RNA was reverse transcribed into cDNA using 500 ng of RNA, which in a final volume of 12 μl supplemented with 1 μl of oligo (dT)_12–18_ (500 μg/ml) and 1 μl of 10 mM dNTP mix, were incubated at 65 °C for 5 min. The mixture was cooled on ice and supplemented with 50 mM Tris-HCl (pH 8.3), 75 mM KCl, 3 mM MgCl_2_, 10 mM DTT, 2 units/μl RNaseOUT Recombinant Ribonuclease Inhibitor (Invitrogen Canada), and 10 units/μl M-MLV RT. Finally, the reverse transcription reaction mixture was incubated at 37 °C for 50 min and heat-inactivated at 70 °C for 15 min. One microliter of the reverse transcription reaction was subsequently used for PCR amplification of β-actin and VPRBP sequences. Briefly, the PCR amplification mix was composed of 20 mM Tris-HCl (pH 8.4), 50 mM KCl, 1.5 mM MgCl_2_, 50 μM dNTPs, 1 μM sense and antisense primers, and 0.05 units/μl Taq DNA polymerase. Primers 5′-GCTCGTCGTCGACAACGG-CTC-3′ and 5′-CAAACATGATCTGGGTCATCTTCTC-3′ were used for β-actin amplification and primers 5′-AGGCCATCCACAAGTTTGAC-3′ and 5′-TCATCTGCCTGCAACATAGC-3′ were used for VPRBP amplification. The PCR amplification conditions for VPRBP were as follows: 94 °C for 2 min; 25 cycles of (94 °C for 45 sec; 57 °C for 30 sec; 72 °C for 1 min); 72 °C for 5 min. The conditions of amplification for β-actin were the same except that 18 cycles of amplification were used.

## Supporting Information

### Accession Numbers

The GenBank (http://www.ncbi.nlm.nih.gov/Genbank/) accession numbers for the proteins discussed in this paper are CUL4A (NM_001008895), DDB1 (NM_001923), and VPRBP (NM_014703).

## Abbreviations

53BP: p53 binding protein
ATM: ataxia telangiectasia mutated
ATR: ataxia telangiectasia mutated and Rad3 related
BRCA1: breast cancer–associated protein
CBP: calmodulin-binding peptide
CUL4A: cullin 4A
DDB1: damaged DNA binding protein 1
DDB2: damaged DNA binding protein 2
GFP: green fluorescent protein
HA: hemagglutinin
HEK: human embryonic kidney
HIV-1: human immunodeficiency virus type 1
RBX1: ring-box 1
siRNA: small interfering RNA
TAP: tandem affinity purification
TEV: tobacco etch virus
UNG2: uracil-DNA glycosylase
Vpr: viral protein R
VPRBP: viral protein R binding protein
VSV-G: vesicular stomatatis virus glycoprotein G
XPC: xeroderma pigmentosum group C protein
